# Microplastic pollution in aquafeed of diverse aquaculture animals

**DOI:** 10.1016/j.heliyon.2024.e37370

**Published:** 2024-09-03

**Authors:** Mohamed Mohsen, Jibin Lin, Kangle Lu, Ling Wang, Chunxiao Zhang

**Affiliations:** a Xiamen Key Laboratory for Feed Quality Testing and Safety Evaluation, Fisheries College, Jimei University, Xiamen City, Fujian Province, 361021, PR China; b Department of Fish Production, Faculty of Agriculture, Al-Azhar University, Nasr City, Cairo, 11884, Egypt

**Keywords:** Contaminants, Ingestion risk, Fiber, Polypropylene, Feed size effect

## Abstract

Microplastics have emerged as pervasive contaminants, and determining their occurrence in aquafeed is key for evaluating their risks to farmed animals and, by extension, humans. However, knowledge about microplastic in aquafeed is still limited. Herein, we determined microplastic characteristics in aquafeed for five important aquaculture animals with different feeding habits. Aquafeed samples were collected for spotted sea bass, shrimp, grass carp, Tilapia, and frogs from main companies in China. The samples were digested using chemical digestion, and the residuals were subjected to a density separation. Microplastics were identified under the microscope and characterized by their shape, color, size, and polymer type. The results showed that microplastics are highly abundant in the feed of frogs, followed by spotted sea bass, Tilapia, grass carp, and shrimp. We found that feed size contributes to the total microplastic abundance in the feed. Further, microplastics were mainly in microfiber form, and the dominant polymer type was propylene, suggesting that packaging and processing are the main sources of pollution. Additionally, the most abundant size of microplastics was 100–1000 μm. Calculating microplastic ingestion risk, the spotted sea bass had the greatest recorded risk of microplastic ingestion, followed by grass carp, frogs, Tilapia, and shrimp. This study lays a foundational step toward understanding microplastic effects on aquaculture animals and calls for further environmentally relevant laboratory experiments to assess the risk of microplastic ingestion on animals and potential transfer to humans.

## Highlights

• Larger feed sizes contain more microplastic.
• Frog feed showed the highest microplastic level.
• Propylene and microfiber are the main microplastics found.
• Spotted sea bass face high ingestion risk.

## Graphical abstract

**Figure:**
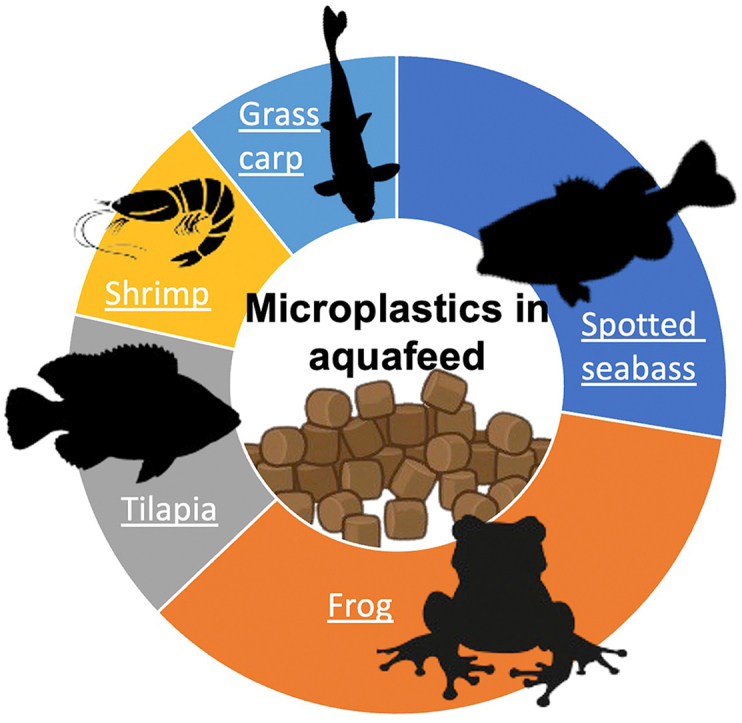

## 1 Introduction

In the last century, the exponential growth of plastic production and utilization has transformed human civilization, revolutionizing industries, enhancing convenience, and enabling technological advancements [1]. However, this proliferation has initiated an unintended consequence - the widespread contamination of the environment with plastic debris [2]. These debris, known as microplastics (≤5 mm), originate from diverse sources, contributing to their pervasive presence in aquatic environments [3]. Primary microplastics are intentionally manufactured small particles used in products like personal care items (microbeads in exfoliants and toothpaste), industrial abrasives, and medical applications. Secondary microplastics result from the fragmentation of larger plastic debris due to environmental degradation processes such as UV radiation, physical abrasion, and microbial action [4].

Because of their widespread sources and persistent nature, microplastics have become ubiquitous in the environment [5]. Once released, microplastics are transported by wind, water currents, and human activities, leading to their accumulation in diverse ecosystems [6,7]. Microplastics are prevalent across marine environments, freshwater systems, terrestrial landscapes, and even the atmosphere [8]. They accumulate in ocean sediments, freshwater bodies, and agricultural soils and have been found in remote locations such as Arctic ice [9]. Additionally, microplastics have infiltrated the human food chain, being detected in seafood, drinking water, and even table salt [10].

The pervasive presence of microplastics extends into aquafeed, a primary source of nutrition for farmed aquatic species, thereby introducing these synthetic particles into the diets of farmed animals [11]. The ingestion of microplastics by these animals might not only affect their health and growth but also pose a risk of transferring these pollutants up the food chain to humans who consume seafood [11,12]. Ingested microplastics might lead to physiological stresses such as impaired growth, reduced reproductive success, and behavioral changes [13]. Also, these contaminants can accumulate in digestive tracts, penetrate tissues, and cause cellular damage, thereby facilitating pathogen spread and antibiotic resistance [13]. Therefore, extending our understanding of microplastic pollution in animal feed is required to evaluate their potential impacts on aquaculture species and, ultimately, human consumers, as these particles can accumulate within the food chain [14]. Also, quantifying microplastics in aquafeed could help to identify the main sources of contamination, inform risk assessment, and guide the development of mitigation strategies to reduce microplastic exposure in aquaculture systems.

Despite many studies concerning microplastic pollution in the ecosystem, knowledge about microplastic pollution in feed is limited, especially in China, the leading country in aquaculture. China holds a pivotal role in global aquaculture production, being the largest producer worldwide and contributing significantly to the supply of various seafood species. Its vast aquaculture industry meets domestic demand and substantially influences the international market, shaping trends and availability across the seafood industry [15]. Aquaculture relies heavily on formulated feed to optimize the growth and health of farmed species [16]. In the feed of European sea bass, 3.9 particles g^−1^ were found [17]. Also, all fish feed samples collected from Bangladesh contained microplastic with an average of 0.5–2.2 particles g^−1^ [18]. Another study from Bangladesh reported that fish feed contained an average of 0.55–11.6 particles g^−1^ [19]. Similarly, a study analyzing feed samples found microplastic contamination ranging from 0.05 to 0.16 items g^−1^ in shrimp feeds, 0.06 to 0.23 items g^−1^ in fish feeds, and 0.09 to 0.33 items g^−1^ in chicken feeds [20].

In the current study, we aimed to characterize microplastics in the feed of animals with different feeding habits, representing omnivorous fish (Tilapia), carnivorous fish (spotted sea bass), and herbivorous fish (grass carp), as well as crustaceans (shrimp) and amphibians (frogs). We further explored the potential ingestion of microplastic by these important farmed species. Comprehensive research is imperative to assess the potential risks posed by microplastics in the diets of farmed animals and to develop sustainable practices mitigating their impact on aquatic life and consumers.

## 2 Materials and methods

### 2.1 Feed samples and chemicals

Different pelleted commercial aquafeeds were collected from feed companies in China. To widen our understanding of microplastic presence in aquaculture feeds and their potential impacts on different aquaculture species, we purchased five commercial feeds representing diverse dietary habits. We included omnivorous fish (Tilapia), carnivorous fish (spotted sea bass), and herbivorous fish (grass carp), as well as crustaceans (shrimp) and amphibians (frogs). These species are commercially valuable and widely farmed aquaculture species consumed by many people worldwide. Batches of 100 g pelleted feed were collected for five farmed animals, with three samples per area. Samples were placed in clean, airtight bags, accurately labeled, shipped in sealed packaging to the laboratory, and kept in airtight containers until inspection. Feed for frogs was collected from Shantou, Zhangzhou, and Chongqing. Feed for Tilapia was collected from Beijing, Chengdu, and Zhangzhou. Feed for grass carp was collected from Jiangmen, Binzhou, and Lianjiang. Feed for shrimp was collected from Zhangzhou, Jieyang, and Chengdu. Feed for spotted sea bass was collected from Foshan and Guangzhou. Zinc chloride was purchased from Macklin Biochemical Technology (Shanghai, China). Hydrogen peroxide was purchased from Xilong Science Company (Shanghai, China). Potassium hydroxide was purchased from Sangon Biotech Company (Shanghai, China).

### 2.2 Microplastic extraction

A sub-sample of 5 g from each feed sample was randomly collected with three replicates. The subsamples were transferred to 500 beakers under a fume hood. These samples were subjected to a previously documented chemical digestion with slight modification [21,22]. The chemical digestion used was a mixture of 180 mL of 10 % KOH and 15 mL of H_2_O_2_ 30 % at 60 °C for 6 h. We applied less amount of H_2_O_2_ (30 %) and less time than previous studies. According to preliminary tests, the digestion was successfully sufficient to dismantle feed pellets compared to using 10 mL of H_2_O_2_ (30 %) or 5 mL (30 %) of H_2_O_2_. However, a floatation test was needed to recover microplastics from the residuals. Therefore, after filtering the digestion solution through an 8 μm filter, ZnCl_2_ solution (1.5 g m^−3^) was added to the residuals. The solution was then stirred for 1 min and kept settling down. Then, the supernatant was filtered through an 8 μm filter paper. All filters were added to a new Petri dish and kept in the fume hood until inspection.

### 2.3 Microplastic characterization

A stereomicroscope was used to examine each Petri dish both horizontally and vertically. Initially, microscopic observation recognized microplastics due to their color and lack of cellular structure. Then, microplastics were subjected to fragmentation resistance when subjected to a tweezer or needle pressure [23]. Recognized particles were kept in a new petri dish and pictured through a fluorescent microscope equipped with a Nikon camera (Tokyo, Japan). The size of the imaged particles was determined using ImageJ software. The particles were kept in a petri dish until polymer-type examination.

### 2.4 Polymer-type examination

Individual microplastic particles were isolated and subjected to Micro-Fourier Transform Infrared Spectroscopy (μ-FTIR) analysis using a specific model stereomicroscope coupled with a Fourier Transform Infrared Spectrometer equipped with relevant accessories. About 50 % of the isolated microplastics were subjected to polymer inspection. Spectra were acquired within a specific spectral range and subsequently analyzed using dedicated software for peak identification and comparison with established spectral libraries. Polymer types were identified by correlating characteristic absorption peaks with reference spectra of known polymers, with matches over 70 % being accepted. A total of 11 particles were identified as non-microplastic particles, including cotton. Quality control measures included analyzing control samples alongside environmental samples and ensuring reproducibility through duplicate or triplicate analyses.

### 2.5 Microplastic contamination control

Controlling contamination throughout the microplastic experimental processes is crucial. All equipment, tools, and laboratory surfaces were meticulously cleaned to mitigate potential contamination. Previously cleaned beakers and handling tools were employed to collect, store, and manipulate samples, minimizing the introduction of extraneous particles. Also, all reagents were filtered through an 8 μm filter paper before use. During sample processing, isolation procedures were conducted in a controlled environment to prevent airborne contamination, often under laminar flow hoods. Stringent protocols, including the use of clean gloves, tweezers, and individualized sample handling, were implemented to avoid cross-contamination between samples. Additionally, blank controls were included in all analyses to monitor and identify potential background contamination from laboratory reagents or handling procedures, ensuring the reliability of results. Regular validation of cleaning procedures and methodological controls was performed to maintain high contamination control standards throughout the entire experimental workflow.

### 2.6 Potential ingestion risk

Calculating the potential ingestion risk is key to assessing the impact of microplastics on the health and growth of aquaculture species. The potential ingestion of microplastics in aquafeed by the five animal species was calculated according to the following formula:

Microplastic ingestion = microplastic abundance × feed intake

Feed intake = feed conversion ration × market weight of the animal

Microplastic abundance represents the number of microplastics in the feed (particles g^−1^). Feed ingested by the farmed animals during the farmed period was calculated based on the feed conversion ratio and the market weight of the animals [14,22]. The feed conversion ratio measures the efficiency with which animals convert feed into body mass. The average feed conversion ratio of the animals used in the current study was reported previously [24,25]. The market size of the animals, on average, was considered 800 g for spotted sea bass, 300 g for frogs, 350 g for Tilapia, 1000 g for grass carp, and 23 g for shrimp.

### 2.7 Statistical analysis

In this study, the normality of data distribution was assessed using the Shapiro-Wilk test to ensure compliance with assumptions underlying parametric statistical tests. The p-values for all groups were greater than 0.05, indicating that the normality assumption was satisfied. Homogeneity of variances was tested using Levene's test, which yielded a p-value of 0.7938, confirming that the variance across groups was homogeneous. Subsequently, a one-way analysis of variance (ANOVA) was employed to determine significant variations among the samples. ANOVA was selected due to its effectiveness in comparing means across multiple groups. Post hoc analysis using the Tukey test was performed to identify specific pairwise differences between groups when ANOVA detected significant differences. Pearson correlation was used to calculate the relationship between feed size and microplastic abundance. All calculations were performed using R software.

## 3 Results and discussion

Determining the number of microplastics in aquafeed is key for assessing the risk of these pollutants, including the potential for physical harm and chemical toxicity to animals, the risk of bioaccumulation and subsequent human exposure to microplastics through the consumption of animal products, and the contribution to environmental pollution. In the current study, we found microplastic in all feed samples. A total number of 33 particles g^−1^ was extracted from all samples, with an average microplastic number of 2.2 particles g^−1^ in all samples, and the highest number of microplastics was found in the feed of frogs and spotted sea bass.

### 3.1 Microplastic amount in aquafeed

**Fig. 1:**
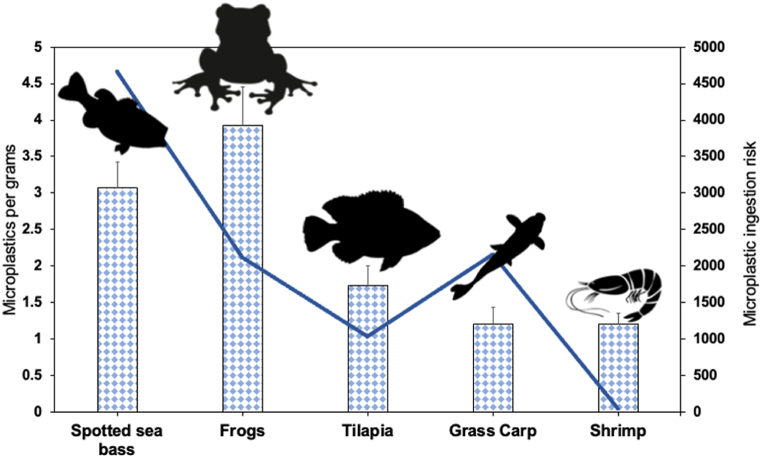
Average microplastic number of every gram of feed for different animals (columns) and potential ingestion risk of microplastics (line). Different letters between the two groups indicate a significant difference (p < 0.05).

The total number of microplastics extracted from all aquafeed samples was 167 particles without detecting microplastics in the blank replicates. The average microplastic number extracted from the feed of spotted sea bass, frogs, Tilapia, grass carp, and shrimp was 3.07, 3.93, 1.73, 1.20, and 1.20 particle g^−1^, respectively (Fig. 1) (microplastics were calculated for every 5 g of feed and converted for every 1 g for comparison with other studies). The range of the total microplastic number from the feed was 0–1.6 particle g^−1^ for spotted sea bass, 0.2–2.6 particle g^−1^ for frogs, 0–1 particle g^−1^ for Tilapia, 0–0.8 particle g^−1^ for grass carp, and 0–0.6 for particle g^−1^ shrimp. Compared to other studies, 3.9 particles g^−1^ were found in the feed of the European sea bass [17], comparable to the number of microplastics found in the feed of the Chinese sea bass in the current evaluation (3.07 particles g^−1^). Furthermore, the average of microplastic particles found (1.2–3.7 particles g^−1^) was comparable to or less than those found in aquafeed from Bangladesh, with an average of 0.5–2.2 particles g^−1^ [18], but less than another study from Bangladesh found an average of 0.55–11.6 particles g^−1^ [19]. Furthermore, microplastics found in the current study were higher than those found in shrimp feeds (0.05–0.16 items g^−1^) and fish feeds (0.06–0.23 items g^−1^) collected from India [20]. This variation is likely due to varied feed ingredients, processing/packaging environment, and feed size.

**Fig. 2:**
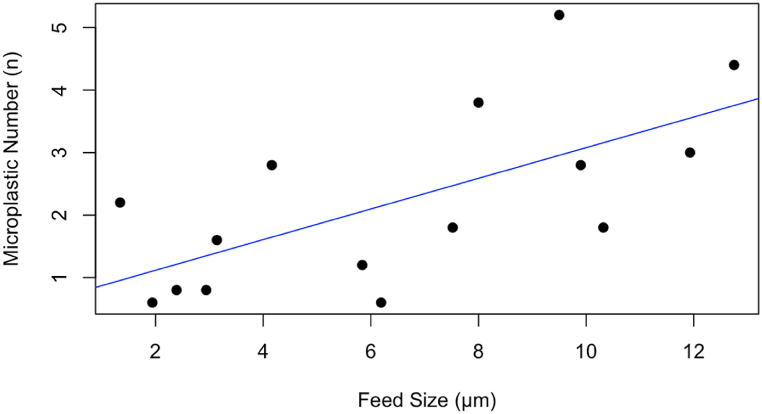
The correlation between the microplastic number and the size of the feed.

Furthermore, to determine whether feed size can influence the number of microplastics in the feed, we calculated the correlation between them. The Pearson correlation coefficient between feed size and the number of microplastics in the feed is approximately 0.6525, with a p-value of 0.0084 (Fig. 2). This indicates a moderate positive correlation, suggesting that as the feed size increases, the number of microplastics in the feed also tends to increase. However, while this correlation is significant, it does not imply causation, and other factors could be influencing the presence of microplastics in animal feed. The highest number of microplastics was found in the feed of frogs and spotted sea bass, likely because of their feed size, which was the largest compared to other feed samples. However, feed ingredients and the processing environment can also contribute to microplastic pollution in the feed. Evidence for these reasons is the moderate positive relationship between feed size and microplastic number, indicating that other factors, such as aquafeed ingredients and the processing environment, could contribute to the microplastic abundance in feed samples [14].

### 3.2 Microplastic characteristics

**Fig. 3:**
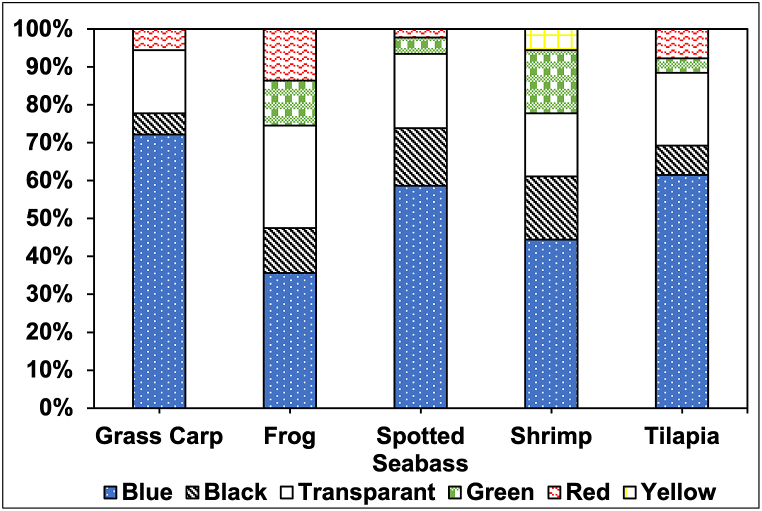
The color variety of microplastics detected in aquafeed samples.

The color of microplastics was mainly blue, followed by transparent, black, green, red, and yellow (Fig. 3). Six colors were found in all feed samples, varying in percentage between different types of feed. This observation aligns with previous studies where similar color distributions of microplastics were found in various feed samples. For instance, blue and transparent microplastics were prominently reported in the feed samples analyzed by Muhib and Rahman (2023), where blue, white, black, transparent, and red were the common colors. Siddique et al. (2023) also identified a similar color spectrum, with white being an additional prominent color not highlighted in the current study. This similarity suggests that blue and transparent microplastics are widespread in aquafeed, potentially due to their common use in packaging and processing materials [17,29].

**Fig. 4:**
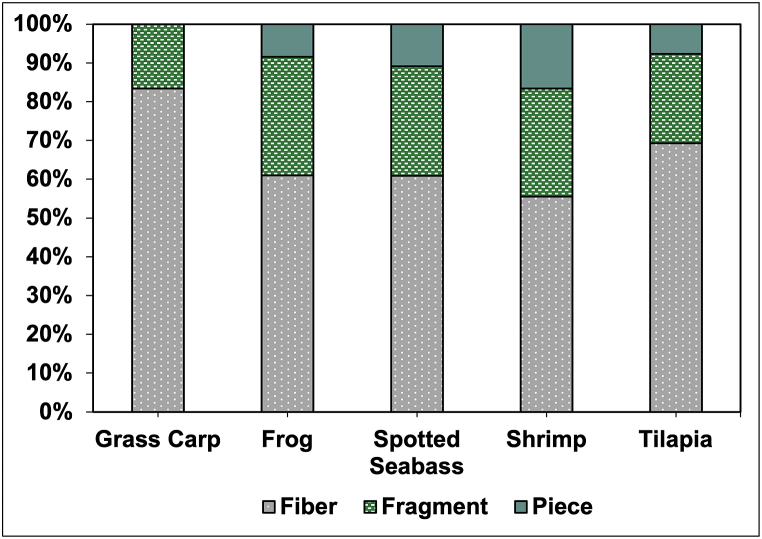
Microplastic types found in aquafeed samples.

For the type of microplastics, microplastic fiber was the highest type of microplastic found in all aquafeed samples (Fig. 4). This is similarly observed from the feed of European sea bass farmed in a recirculating aquaculture system [17]. Microfibers were also dominant in fish feed samples collected from Bangladesh [18]. These microfibers are recognized as a predominant form of microplastics detected across various global environmental samples, from the deepest oceans to remote mountainous regions [26]. Microfibers are considered hazardous mainly due to their shape, which allows them to be easily ingested by aquatic and terrestrial organisms, potentially leading to physical blockages and chemical toxicity [27]. These fibers can absorb and carry pollutants, such as heavy metals [28], introducing these harmful substances into the food chain.

**Fig. 5:**
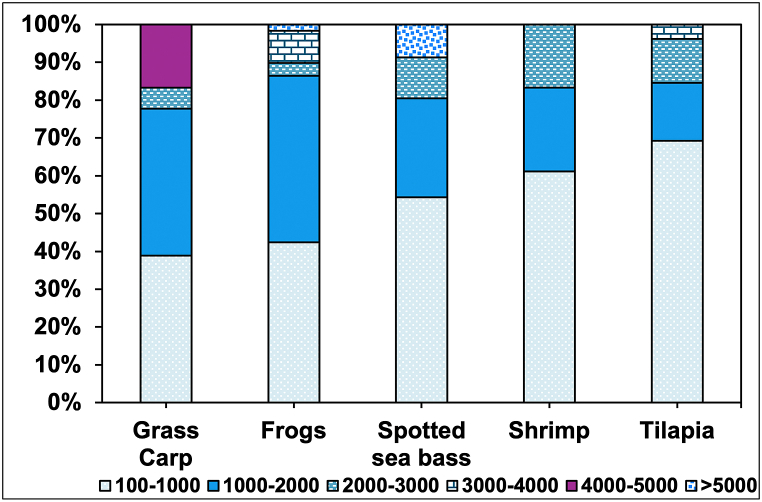
The size of microplastics extracted from aquafeed samples.

The size of microplastics ranged from 113 to 7602 μm, with an average of 1294 μm. The most abundant size was 100–1000 μm microplastics, and a few numbers exceeded 5 μm (Fig. 5). This size distribution overlaps with previous findings in different studies, indicating a similar range of microplastic sizes in the feed samples. Siddique et al. [18] reported microplastic sizes ranging from 13 μm to 5000 μm studies, and 100–1500 μm size was highly abundant. Also, Muhib and Rahman [19] reported microplastics in the feed samples ranging from 14 μm to 4480 μm. The significant presence of microplastics in the 100–1000 μm range is noteworthy as it indicates the potential for these particles to be ingested by a wide range of farmed species, posing health risks and potential bioaccumulation [3].

### 3.3 Microplastic polymer type

**Fig. 6:**
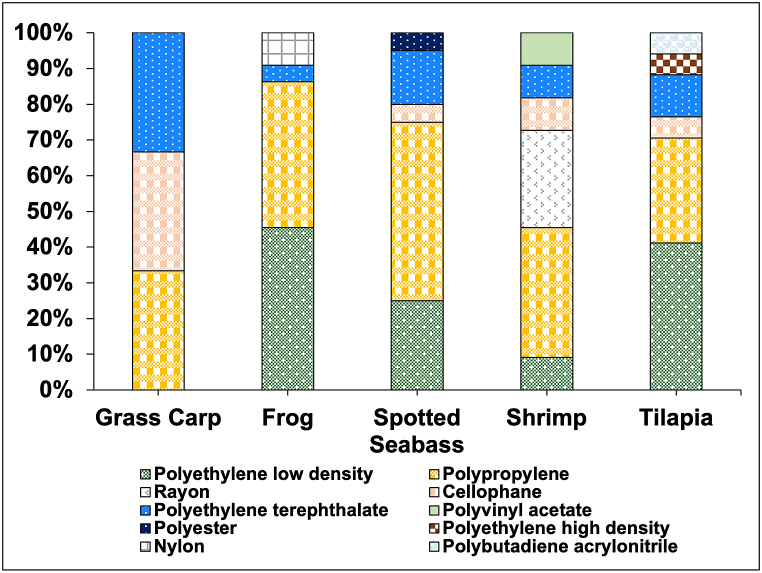
Polymer-type composition of microplastics extracted from feed samples.

**Fig. 7:**
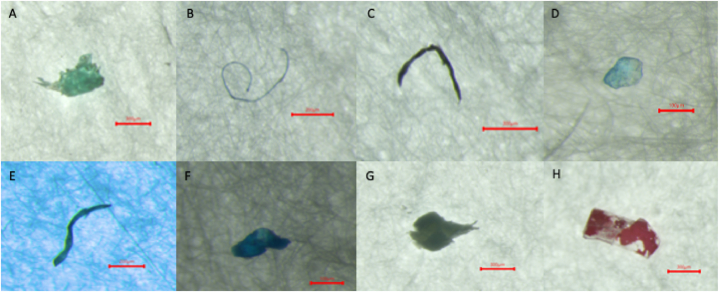
Examples of microplastic shapes and colors and their identified polymers. A: a green piece of microplastic identified as polyethylene low density (scale: 300 μm); B: a blue fiber of microplastic identified as polyethylene terephthalate (scale: 200 μm); C: a black fragment microplastic identified as polypropylene (scale: 300 μm); D: a blue piece of microplastic identified as polyethylene low density (scale: 100 μm); E: black fragment microplastic identified as polypropylene (scale: 200 μm). F: a blue piece of microplastic identified as polyethylene low density (scale: 100 μm); G: a green piece of microplastic identified as polypropylene (scale: 300 μm); H: a red piece of microplastic identified as polypropylene (scale: 300 μm).

Ten polymer types were identified for microplastics isolated from aquafeed samples (Fig. 6). Various polymers were identified from different shapes and colors (Fig. 7A–H). Three polymers were identified in the feed of seagrass carp, including polypropylene, cellophane, and polyethylene terephthalate. In frog feed, 4 polymers were identified, including polypropylene, polyethylene low density, polyethylene terephthalate, and nylon. In spotted sea bass feed, 5 polymers were identified, including polypropylene, polyethylene low density, polyethylene terephthalate, cellophane, and polyester. For shrimp feed, 6 polymers were detected, including polypropylene, rayon, polyethylene low-density, cellophane, polyethylene terephthalate, and polyvinyl acetate. In the Tilapia feed, 6 different polymers were identified, including polyethylene low density, polypropylene, polyethylene terephthalate, cellophane, polyethylene high density, and polybutadiene acrylonitrile.

Polypropylene was the predominant microplastic type found in the current study, which is similarly reported from aquafeed samples collected from Bangladesh [19]. However, propylene was not the major polymer type found in other studies that collected feed samples from Portugal [17] and Bangladesh [18]. This variation is likely due to different pollution sources in feed ingredients or processing and packaging environments. Polypropylene microplastics in environmental samples primarily originate from the breakdown of larger polypropylene items used in various consumer and industrial applications [30]. These include packaging materials, textiles, automotive parts, and household goods. These items can degrade into smaller fragments during use and disposal, releasing microfibers into the environment.

### 3.4 Potential ingestion risk

In the current study, we calculated the potential ingestion of microplastics by multiplying the number of microplastics found per gram and the feed intake of the five animals. This ingestion risk might vary greatly according to feed size, processing and packaging environment, and feed intake. The potential ingestion risk of microplastics for the five farmed animals was 4661, 2160, 2122, 1029, and 47 for spotted sea bass, grass carp, frogs, Tilapia, and shrimp, respectively. The highest value of microplastic ingestion risk was recorded for spotted sea bass, followed by grass carp, frogs, Tilapia, and shrimp (Table 1). This was because of the high number of microplastics found and the feed intake amount. It was found that Tilapia could potentially consume 225 microplastics from contaminated fishmeal until reaching a weight of 250 g [22]. Also, shrimp may consume 37 microplastic until reaching 18 g [22]. These ingestion risks are lower than the potential ingestion risk reported in the current study, which is likely because the feed contains ingredients other than fishmeal that can contribute to the potential ingestion risk through feed in addition to the processing and packaging environment [14].

**Table 1:** Microplastic ingestion risk by the five aquaculture animals.

Species	AVG microplastics (n)	AVG FCR	AVG market weight (g)	Feed intake (g)	Plastic ingestion risk	References
Frogs	3.93	1.8	300	540	2122	[24]
Sea bass	3.07	1.9	800	1520	4661	[25]
Tilapia	1.73	1.7	350	595	1029	
Shrimp	1.20	1.7	23	39.1	47	
Grass carp	1.20	1.8	1000	1800	2160	

AVG: Average.

### 3.5 Laboratory studies realism in microplastic effects on aquaculture animals

It is crucial to investigate the impact of microplastics on aquaculture animals because of the increasing dependence on aquaculture for food production and its significance in global food security. This information is essential for protecting the health and well-being of aquaculture species and guaranteeing the quality and safety of aquaculture products for human use. For instance, exposure to polypropylene microplastic (500 and 5000 μg/L) in the spotted sea bass led to accumulation in spleen and kidney tissues, cytotoxicity, and weakened antiviral response. This impaired the immune system, increased virus replication, and lowered immune gene expression in tissues, raising the susceptibility to viral diseases [31]. Also, Nile tilapia exposed to dietary ingestion of propylene microplastics with concentrations of 100 and 500 μg of polypropylene/kg for 30 d showed adverse consequences. These effects manifested as systemic inflammatory disturbances attributed to liver function alterations, leukocyte profile, organ morphometry, and changes in the intestinal flora [32]. Additionally, grass carp exposed to 100 and 500 μg L polystyrene microplastics resulted in a noticeable reduction in growth rate and alteration in oxidative stress and intestinal microbial community [33]. Furthermore, dietary exposure to 40 and 400 μg polystyrene microplastics/kg resulted in oxidative stress and histological damage [34]. Moreover, ingesting polyethylene microplastics has sublethal consequences on the growth and development of frogs [35]. However, these microplastic effects need further illustrations regarding microplastic shape and concentrations to be compatible with microplastic characteristics in the environmental samples.

When reporting the effects of microplastics, it is crucial to use environmentally relevant concentrations and characteristics of microplastics in laboratory studies to report their effects on aquaculture species accurately. It has been reported that most of the early experimental research on the effect of microplastics on organisms, which stated a severe environmental risk, used very high microplastic concentrations not relevant to most/all environments [36]. Only 17 % of 89 experimental studies on microplastic effects used natural concentrations, while 80 % used a lower microplastic size than most environmental samples [36]. Furthermore, different microplastic characteristics might lead to different toxicity and physiological responses. For instance, fibers may cause more damage than other shapes [37]. However, laboratory studies often use microbeads/fragments, which complicates the interpretation of laboratory findings in a real-world context [38]. Thus, there is a need for more environmentally realistic studies concerning microfiber concentrations reported from the field, as well as their associated pollutants, to understand their risk on farmed animals. Also, the color of microplastics can affect their visibility to aquatic organisms [39]⁠ and, thus, influence ingestion rates and subsequent biological effects. Additionally, given the small size of the detected microplastics, there is a potential for these particles to translocate into animal tissues and transfer through the food chain [40,41]. This bioaccumulation and potential biomagnification pose serious health risks, as microplastics can carry persistent organic pollutants (POPs) and other toxic substances [42].

Thus, the current report provides essential knowledge for determining the effects of microplastics on five important aquaculture species. Microplastic characteristics reported herein, including concentrations, shapes, and sizes, should be tested in laboratory studies.

## 4 Conclusion

The current study investigates microplastics within aquafeed across five significant aquaculture species in China. This research lays a foundational step towards understanding and addressing microplastic pollution in aquaculture, advocating for sustainable practices that ensure the health of aquatic ecosystems and the safety of our food supply. The results indicated that the feed size is another factor contributing to the amount of microplastics in aquafeed. The microplastic amount was highest in frog feed, followed by spotted seabass, Tilapia, grass carp, and shrimp. Furthermore, microplastic fiber was dominant across the recorded microplastic shapes, and polypropylene was the major polymer type. The knowledge reported herein is important for determining the potential effects of microplastics on the tested species. Calculating the potential ingestion risk, the highest microplastic ingestion was recorded for spotted sea bass, followed by grass carp, frogs, Tilapia, and shrimp. Further studies should use the information reported herein in laboratory studies to examine the effects of microplastics.

## Data availability statement

Data will be made available on request.

## CRediT authorship contribution statement

**Mohamed Mohsen:** Writing – original draft, Data curation, Conceptualization. **Jibin Lin:** Investigation. **Kangle Lu:** Writing – review & editing. **Ling Wang:** Writing – review & editing. **Chunxiao Zhang:** Writing – review & editing.

## Declaration of competing interest

The authors declare that they have no known competing financial interests or personal relationships that could have appeared to influence the work reported in this paper.
