# Supplementary figures and images for: Microplastic pollution in aquafeed of diverse aquaculture animals
Source: Heliyon. 2024 Sep 3;10(17):e37370. doi: 10.1016/j.heliyon.2024.e37370 (PMC11408773; doi:10.1016/j.heliyon.2024.e37370)

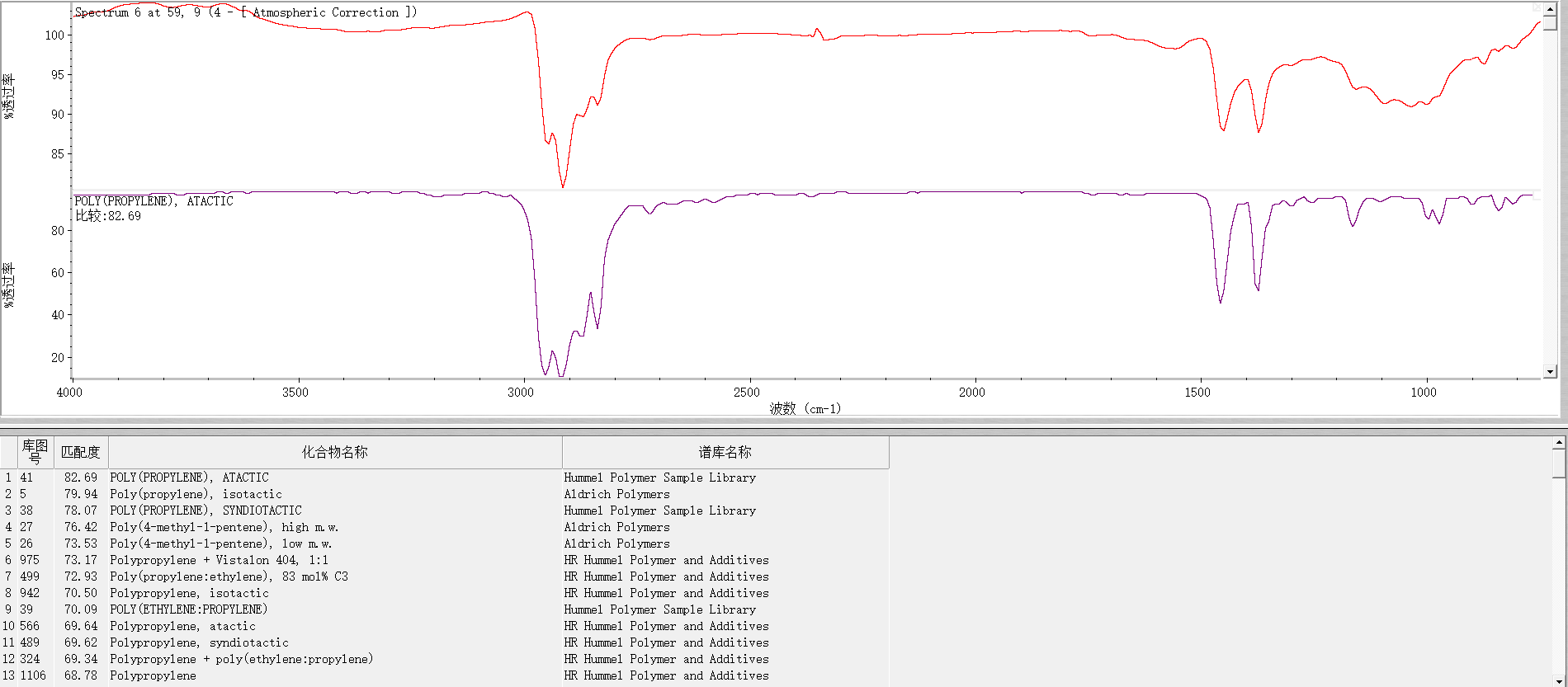

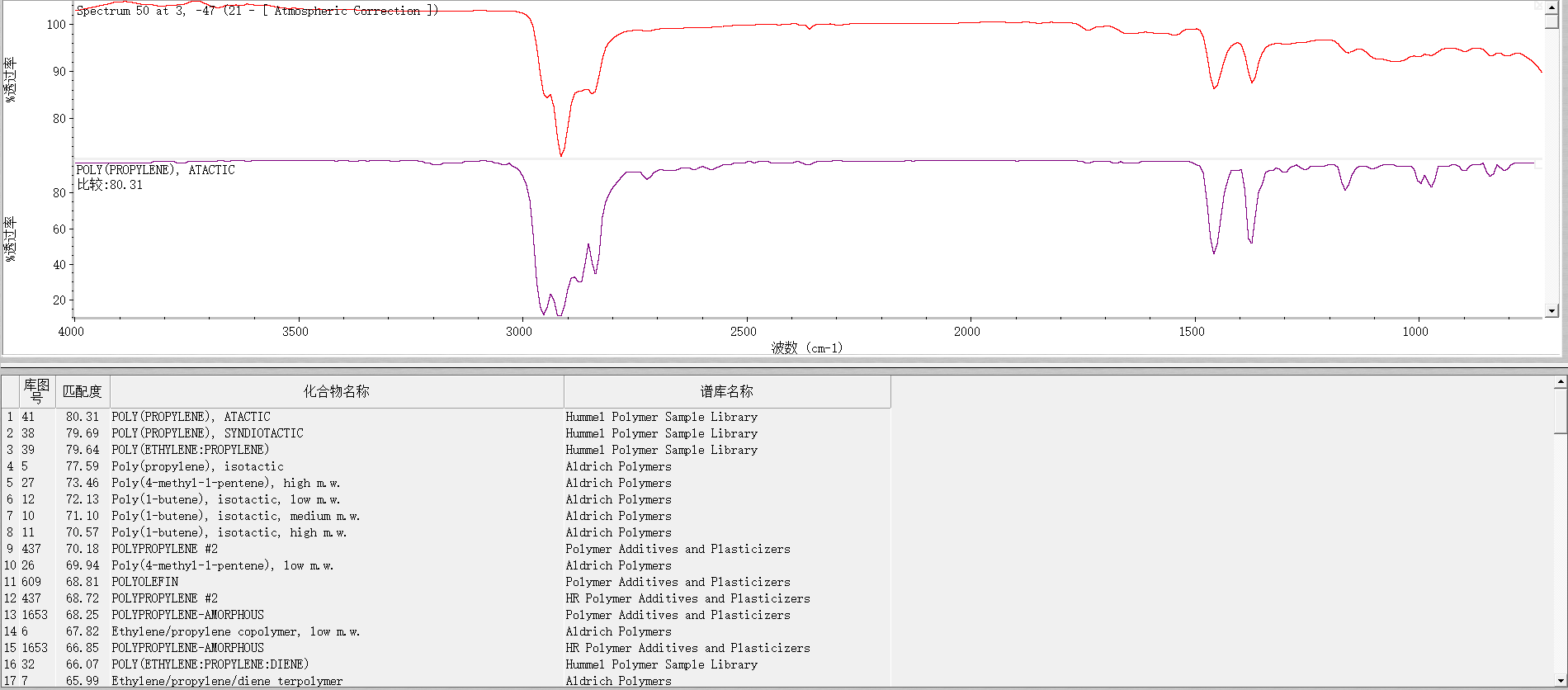

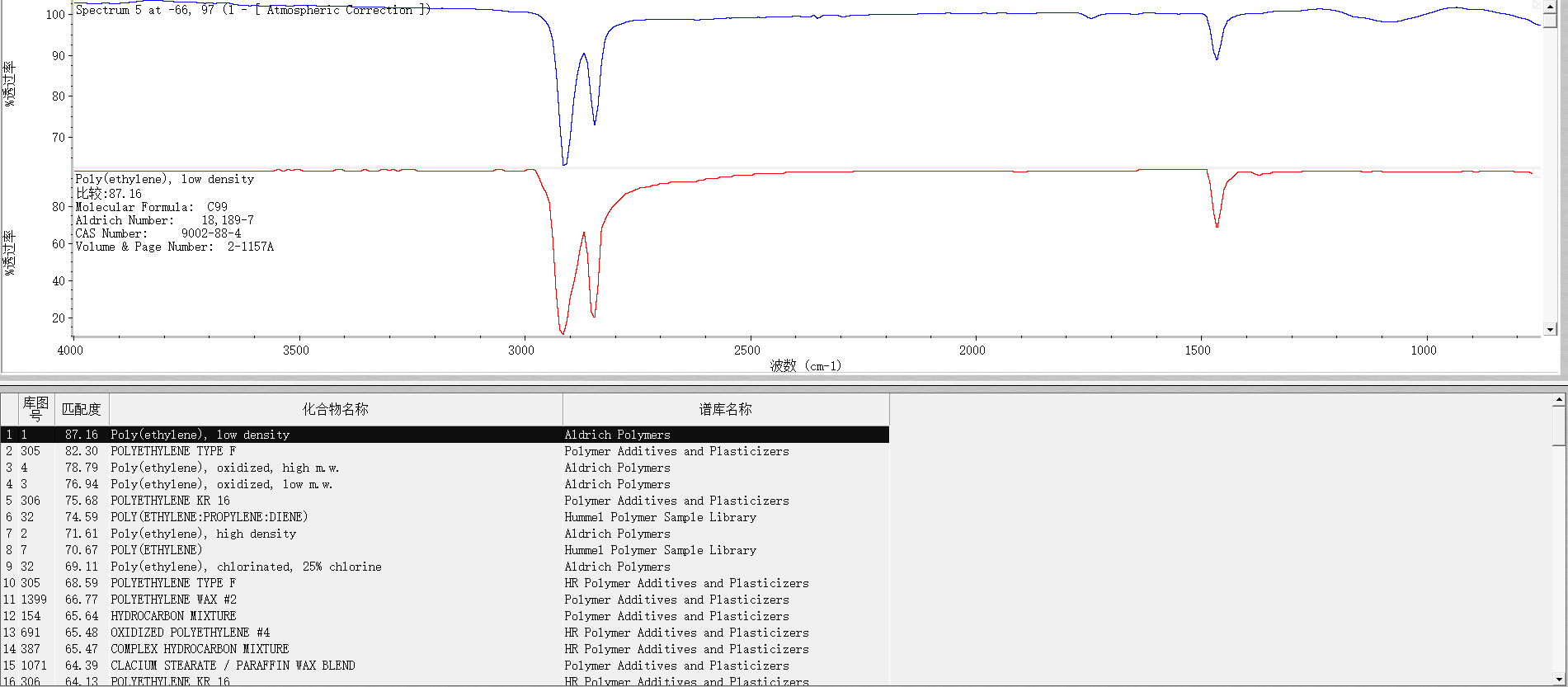
Examples of FTIR library matching results


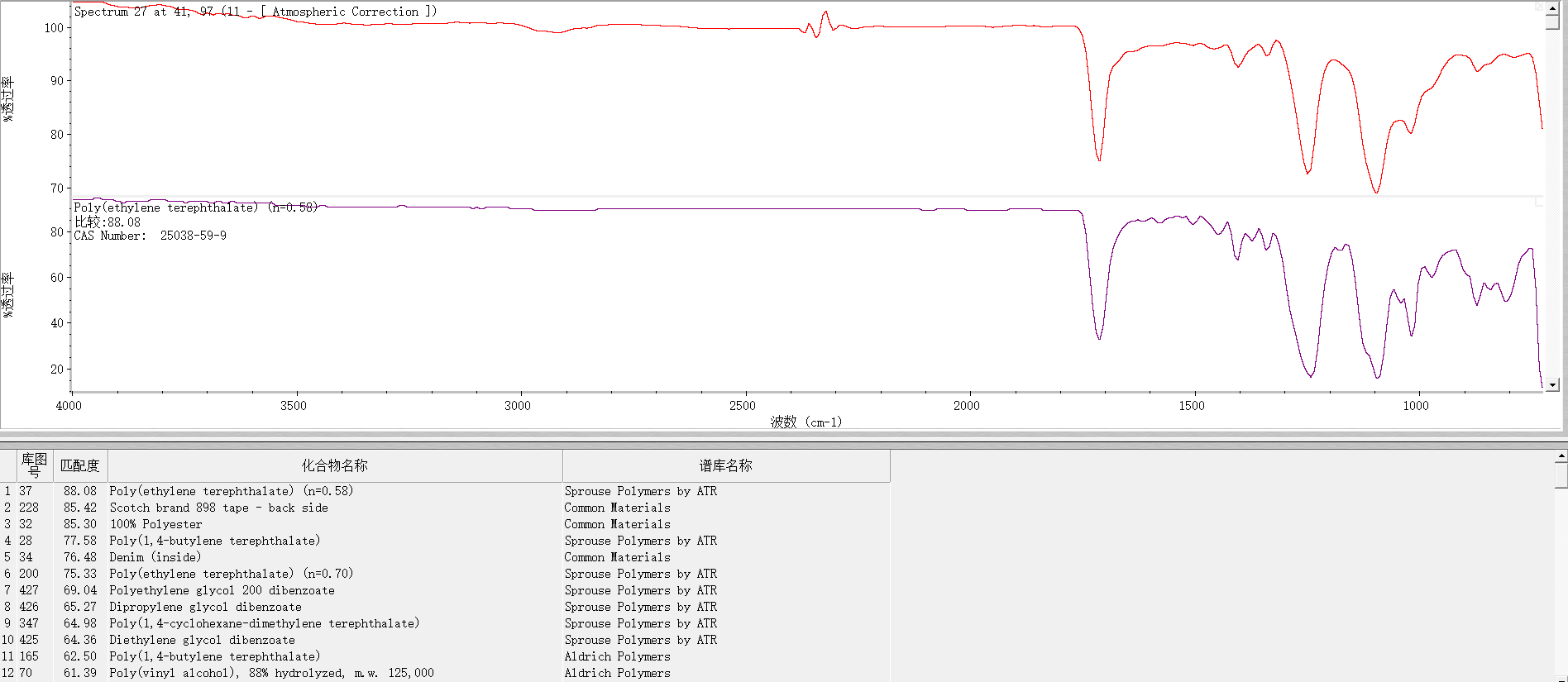

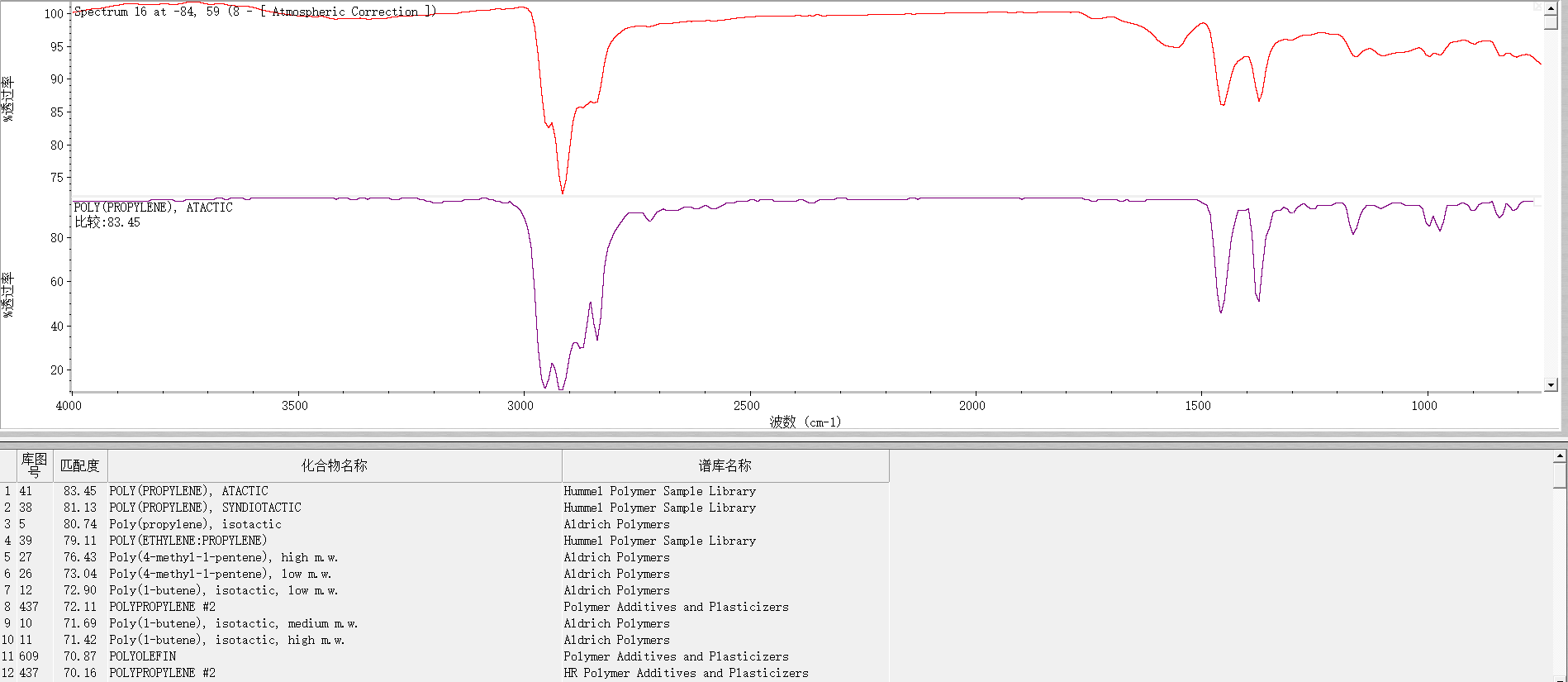

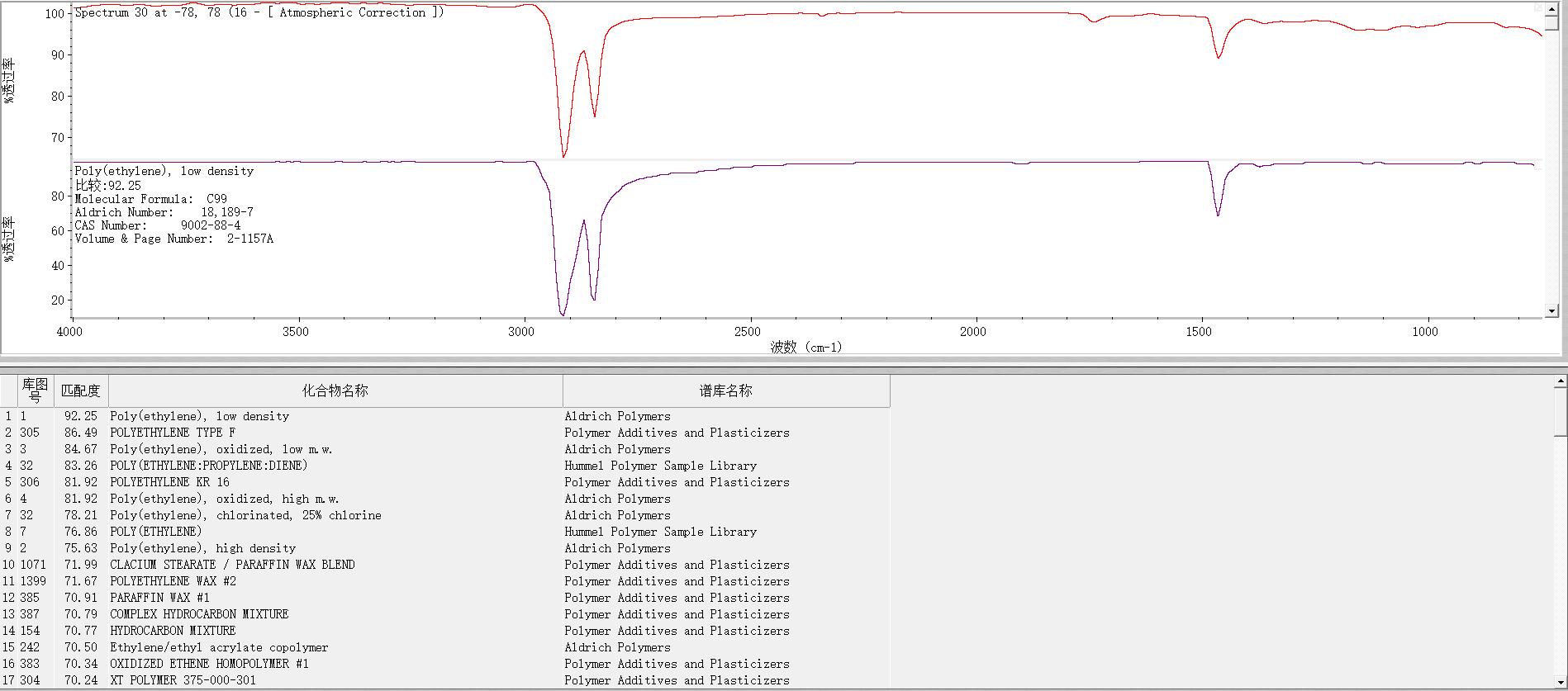

Supplement: Multimedia component 1 [file mmc1.docx]
